# Gut Microbiome Signatures in the Progression of Hepatitis B Virus-Induced Liver Disease

**DOI:** 10.3389/fmicb.2022.916061

**Published:** 2022-06-06

**Authors:** Ranxi Li, Xinzhu Yi, Junhao Yang, Zhou Zhu, Yifei Wang, Xiaomin Liu, Xili Huang, Yu Wan, Xihua Fu, Wensheng Shu, Wenjie Zhang, Zhang Wang

**Affiliations:** ^1^South China Normal University-Panyu Central Hospital Joint Laboratory of Basic and Translational Medical Research, Guangzhou Panyu Central Hospital, Guangzhou, China; ^2^School of Life Sciences, South China Normal University, Guangzhou, China; ^3^Department of Gastroenterology, Guangzhou Panyu Central Hospital, Guangzhou, China; ^4^Department of Infectious Diseases, Guangzhou Panyu Central Hospital, Guangzhou, China; ^5^Department of Science and Education, Guangzhou Panyu Central Hospital, Guangzhou, China

**Keywords:** hepatitis, liver cirrhosis, hepatocellular carcinoma, hepatitis B virus, meta-analysis, gut microbiome

## Abstract

The gut microbiome is associated with hepatitis B virus (HBV)-induced liver disease, which progresses from chronic hepatitis B, to liver cirrhosis, and eventually to hepatocellular carcinoma. Studies have analyzed the gut microbiome at each stage of HBV-induced liver diseases, but a consensus has not been reached on the microbial signatures across these stages. Here, we conducted by a systematic meta-analysis of 486 fecal samples from publicly available 16S rRNA gene datasets across all disease stages, and validated the results by a gut microbiome characterization on an independent cohort of 15 controls, 23 chronic hepatitis B, 20 liver cirrhosis, and 22 hepatocellular carcinoma patients. The integrative analyses revealed 13 genera consistently altered at each of the disease stages both in public and validation datasets, suggesting highly robust microbiome signatures. Specifically, *Colidextribacter* and *Monoglobus* were enriched in healthy controls. An unclassified *Lachnospiraceae* genus was specifically elevated in chronic hepatitis B, whereas *Bilophia* was depleted. *Prevotella and Oscillibacter* were depleted in liver cirrhosis. And *Coprococcus* and *Faecalibacterium* were depleted in hepatocellular carcinoma. Classifiers established using these 13 genera showed diagnostic power across all disease stages in a cross-validation between public and validation datasets (AUC = 0.65–0.832). The identified microbial taxonomy serves as non-invasive biomarkers for monitoring the progression of HBV-induced liver disease, and may contribute to microbiome-based therapies.

## Introduction

Hepatitis B virus (HBV) infection is the leading cause of liver-related deaths in the Asia-Pacific region (Sarin et al., 2020). HBV infection can be divided into several clinical stages according to the extent of liver injury: (1) chronic hepatitis B (CHB), diagnosed as chronic HBV infection, and could be asymptomatic or cause a chronic inflammation of the liver; (2) liver cirrhosis (LC), which is the impaired liver function caused by fibrosis, leading to high morbidity and mortality (Xiao et al., 2019); (3) hepatocellular carcinoma (HCC), the most common cause of death in patients with liver cirrhosis. HCC develops in the setting of chronic liver inflammation and is characterized by constant cycle of damage caused by immune system repeatedly attacking the liver cells due to chronic HBV infections.

The liver and gut originate from the same germ layer and have anatomical and functional connections, known as the “gut-liver” axis (Marshall, 1998). Bile secretion and hepatic portal systems play a crucial role in the interaction between the liver and the gut (Cesaro et al., 2011). Bile produced in the liver is carried by the bile ducts to the connected intestine, and almost all circulating blood from the gut must pass through the liver. Increasing evidence demonstrates an association between gut microbiota and HBV-induced liver diseases. For instance, in CHB patients, *Anaerostipes*is enriched where as potential beneficial taxa such as *Bifidobacterium* is significantly decreased (Xu et al., 2012; Yun et al., 2019). Potential gut pathogens such as those from *Enterobacteriaceae* are highly abundant in the gut microbiome of liver cirrhosis patients, whereas some short-chain fatty acid producers such as *Lachnospiraceae* and *Ruminococcaceae* are decreased (Wong et al., 2006; Chen et al., 2011; Lepage et al., 2011; Flint et al., 2012). Microbial translocation and TLR4 signaling was involved in the development of HCC, in which *Ruminococcus, Coprococcus, Subdoligranulum*, and *Clostridium IV* capable of producing butyrate were decreased (Holmstrom et al., 2004; Dapito et al., 2012; Mangifesta et al., 2018; Ren et al., 2019). Most of these microbiome studies focused on one particular stage of HBV-induced liver disease against another, while a comprehensive view of microbiome alterations along disease progression is lacking.

With increasing availability of public microbiome datasets, it is possible to interrogate diseases-associated microbiome alterations through meta-analysis. By synthesizing from multiple datasets with enhanced signal-to-noise ratio, microbiome meta-analysis is powerful in identifying highly robust disease-related microbial signatures (Pammi et al., 2017; Wang et al., 2020; Wu et al., 2021). In this study, we systematically collected 16S rRNA gene sequencing-based gut microbiome datasets involving CHB, LC and HCC. A standardized pipeline was established to analyze each dataset and the microbial signatures were synthesized across studies using a random effect statistical meta-analysis. Together with an independent cohort validation, we identified robust signatures of the gut microbiome in association with the progression of HBV-infected liver disease.

## Materials and Methods

### Collection of Microbiome Datasets

**Figure 1:**
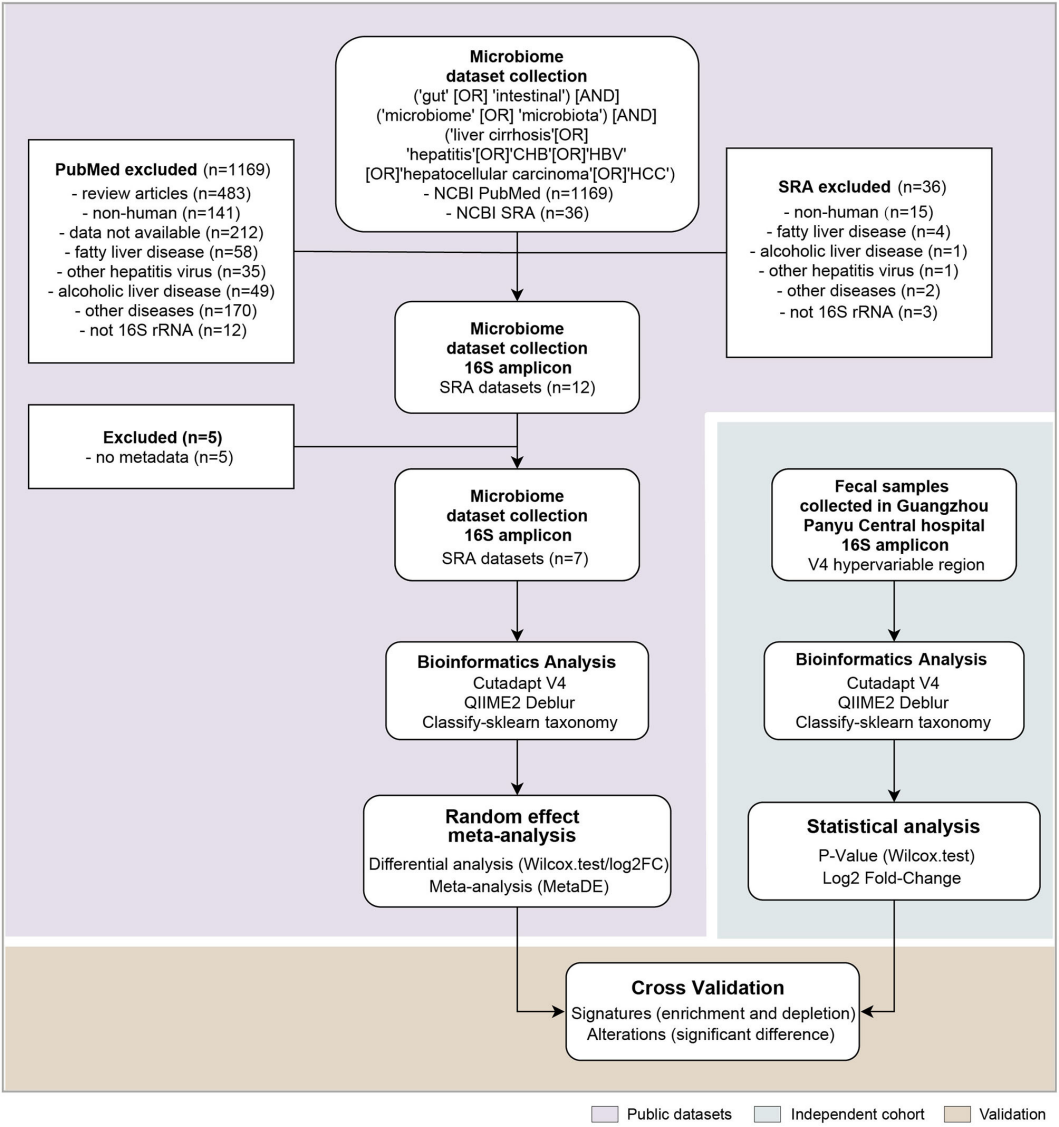
The analysis pipeline in this study, such as the flowchart of the meta-analysis for gut microbiome of public datasets, and the independent cohort validation. Each step is shown in the white box, and the statistical methods and software used are within. *n* denotes the number of datasets included in each step.

All public 16S rRNA gene gut microbiome datasets were retrieved from the National Center for Biotechnology Information by searching for publications in PubMed and searching for datasets in Sequence Read Archive that contained at least 1 of the words “microbiota” (OR) “microbiome” with the term “gut” (OR) “intestinal”, as well as “CHB” (OR) “hepatitis B” (OR) “HBV” for chronic hepatitis B microbiome samples, “liver cirrhosis” for liver cirrhosis ones, “hepatocellular carcinoma” (OR) “HCC” for hepatocellular carcinoma anywhere in the article or in BioProjects (for possibly unpublished data). We excluded fatty liver disease, alcoholic liver disease, other hepatitis virus-caused, and other hepatology not concerned with cirrhosis in the search results. Also, non-human studies and review articles were removed (Supplementary Table S1). The remaining articles had metadata with clear annotation on disease status (Figure 1; Table 1).

**Table 1:** Summary of the public datasets included in the meta-analysis.

**BioProject**	**PMID**	**Region**	**Platform**	**16s region**	**PE vs. SE**	**Samples**			
						**Healthy control**	**CHB**	**HBV-LC**	**HBV-HCC**
PRJNA558158	32265857	Xiamen, China	Illumina HiSeq 2500	V3–V4	PE	21	28	25	
PRJNA382861	29180991	Shanghai, China	Illumina MiSeq	V3–V4	PE	22	85		
PRJNA445763	29780327	Harbin, China	Illumina HiSeq 2500	V3–V4	PE	20		30	
PRJNA540574	32281295	Jilin, China	Illumina HiSeq 2500	V4	PE	20	8	8	35
PRJEB32568	NA	Yantai, China	Illumina HiSeq 2500	V4–V5	SE	5	12	11	9
PRJNA428932	30675188	Nanjing, China	Illumina TruSeq	V4	SE	33			35
PRJNA478823	31293562	Guangzhou, China	Illumina MiSeq	V4–V5	SE	18			61

### Data Retrieval and Processing

The raw sequencing data of public datasets were downloaded from Sequence Retrieve Archive, European Nucleotide Archive, or from links provided in publications. All 16S rRNA gene sequencing data were processed using QIIME2 platform (Bolyen et al., 2019). For each dataset, the pair-end sequences were assembled and merged by FLASH (Magoc and Salzberg, 2011) under the parameters with -M 150. All sequences were truncated to the V4 hypervariable region by Cutadapt (Kechin et al., 2017) under the parameters with -g GTGYCAGCMGCCGCGGTAA (515F) and -a ATTAGAWACCCBNGTAGTCC (806R). The trimmed sequencing reads were denoised to generate a to generate amplicon sequence variants (ASVs) using Deblur (Amir et al., 2017; Nearing et al., 2018), followed by taxonomy assignment with classify-sklearn based on Naive Bayes model (Bokulich et al., 2018), using a classifier built on the V4 rRNA sequences from SILVA database (silva-138-99-515-806-nb-classified.qza) (Yilmaz et al., 2014). Sequences belonging to mitochondria, chloroplast and unclassified or singleton ASVs were excluded. The feature tables were rarefied at the frequency of the minimum reads within a dataset.

### Recruitment of Participants

Patients' recruitment and diagnosis were made by experienced physicians at Guangzhou Panyu Central Hospital, Guangzhou, China, from October 2020 to July 2021. The diagnoses of CHB, LC, and HCC were made based on iconography examination, positive pathological examinations, viral serologic testing, or chronic liver disease background. Patients with severe complications, such as gastrointestinal bleeding, spontaneous bacterial peritonitis, and history of radiotherapy and chemotherapy within a year of diagnosis were excluded. The healthy control group consisted of healthy volunteers from Guangzhou Panyu Central Hospital who met inclusion criteria of (a) 18 years or older; (b) no clinical or biochemical evidence of intestinal and liver-related diseases that of HBV infection; and (c) absence of regular or excessive use of alcohol. All participants were filtered by the exclusion criteria of (a) history of antibiotic, microecological preparation, or immunosuppressant treatment within the past 4 weeks; (b) diagnosis of diabetes mellitus, autoimmune disease, such as multiple sclerosis, rheumatoid arthritis, hypertension, coronary heart disease, or metabolic syndrome; and (c) pregnancy or lactation. Written Informed consent was obtained from all participants.

### Sampling, DNA Extraction and PCR

Fresh tail stool samples of more than 0.5 g were collected from all participants with informed consent at hospital, frozen at −80°C within an hour, and stored until use. DNA was extracted from each sample and purified with the MagaBio Soil/Feces Genomic DNA Purification Kit (Hangzhou Bioer Technology Co. Ltd.). The concentration and purity of isolated DNA were detected with Thermo NanoDrop One (Thermo Fisher Scientific, MA, USA). PCR was measured in BioRad S1000, targeting the V4 hypervariable region of the bacterial 16S rRNA gene with the forward primer 341F (5′-CCTAYGGGRBGCASCAG-3′) and the reverse primer 806R (5′-GGACTACNNGGGTATCTAAT-3′). Sequencing library was constructed with NEBNext Ultra II DNA Library Prep Kit for Illumina^®^ (New England Biolabs, MA, USA) according to the manufacturer's instruction and then assessed on the Qubit@ 2.0 Fluorometer (Thermo Fisher Scientific, MA, USA). Pair-end reads (250 bp × 2) were performed by the Guangdong Magigene Biotechnology Co. Ltd. (Guangzhou, China) on an Illumina Nova6000 platform (Illumina Inc., CA, USA).

### Statistical Analyses

The genus-level taxa with an average relative abundance >0.01% in all seven public datasets were retained. To address compositionality, the microbiome relative abundance data were arcsine-square root-transformed and *z*-score-normalized before assessed for differentially abundant taxa using Wilcoxon rank-sum test. The summary statistics between studies were integrated according to six pairwise comparisons such as Healthy vs. CHB, Healthy vs. LC, Healthy vs. HCC, CHB vs. LC, CHB vs. HCC, and LC vs. HCC, by a random effect statistical meta-analysis using the MetaDE v1.0.5 package in R v3.6.1 (Wang et al., 2012). The effect size combination method was chosen for meta-analysis since it generates more conservative and biologically consistent results than does the *p*-value combination method (Marot et al., 2009). The procedures for sequencing data processing and differential taxonomic analysis on the validation dataset are identical to those for the public datasets. The microbiome of the validation dataset was rarefied to 33,122 reads per sample. The genera with *p* < 0.05 and consistent direction of alteration in both public and validation datasets were retained.

To test the diagnostic capabilities of the identified microbial taxa, webuilt LASSO logistic regression models between each pairwise comparison of health and disease stages and selected microbial taxa using glmnet R package (Tibshirani, 1996; Friedman et al., 2010). To test the generalizability of the models, each model was cross-validated between public and validation datasets. To alleviate the impact of the heterogeneity of different public datasets, for each comparison, the model was trained using one public dataset selected according to the sample size and its balance between the two groups, and tested in the validation cohort (Supplementary Table S2). The hyperparameter of LASSO was optimized for each classifier in a nested 5-fold cross-validation within the training subset. ROC curves were plotted using the pROC package in R (Robin et al., 2011).

## Results

The strategy of our analysis is shown in Figure 1. PubMed and Sequence Retrieve Archive (SRA), searched with the joint query (see Methods), returned 1,169 citations and 36 accessions in BioProjects, respectively. After excluding studies on fatty liver disease, alcoholic liver disease, non-HBV caused liver disease and liver disease unrelated to cirrhosis, twelve16S rRNA gene datasets remained (Supplementary Table S1). Total five datasets were excluded due to missing metadata information, resulting in seven 16S rRNA gene datasets such as 139 healthy controls, 133 CHB,74 LC, and 140 HCC samples for meta-analysis (Table 1). Different hypervariable regions were shown to have a significant impact for microbiome meta-analysis (Wang et al., 2020). Despite that the targeted hypervariable region of the 16S rRNA gene varied across studies, they all covered the V4 region. For consistency, we truncated sequences from all studies to V4 region prior to downstream analyses.

**Figure 2:**
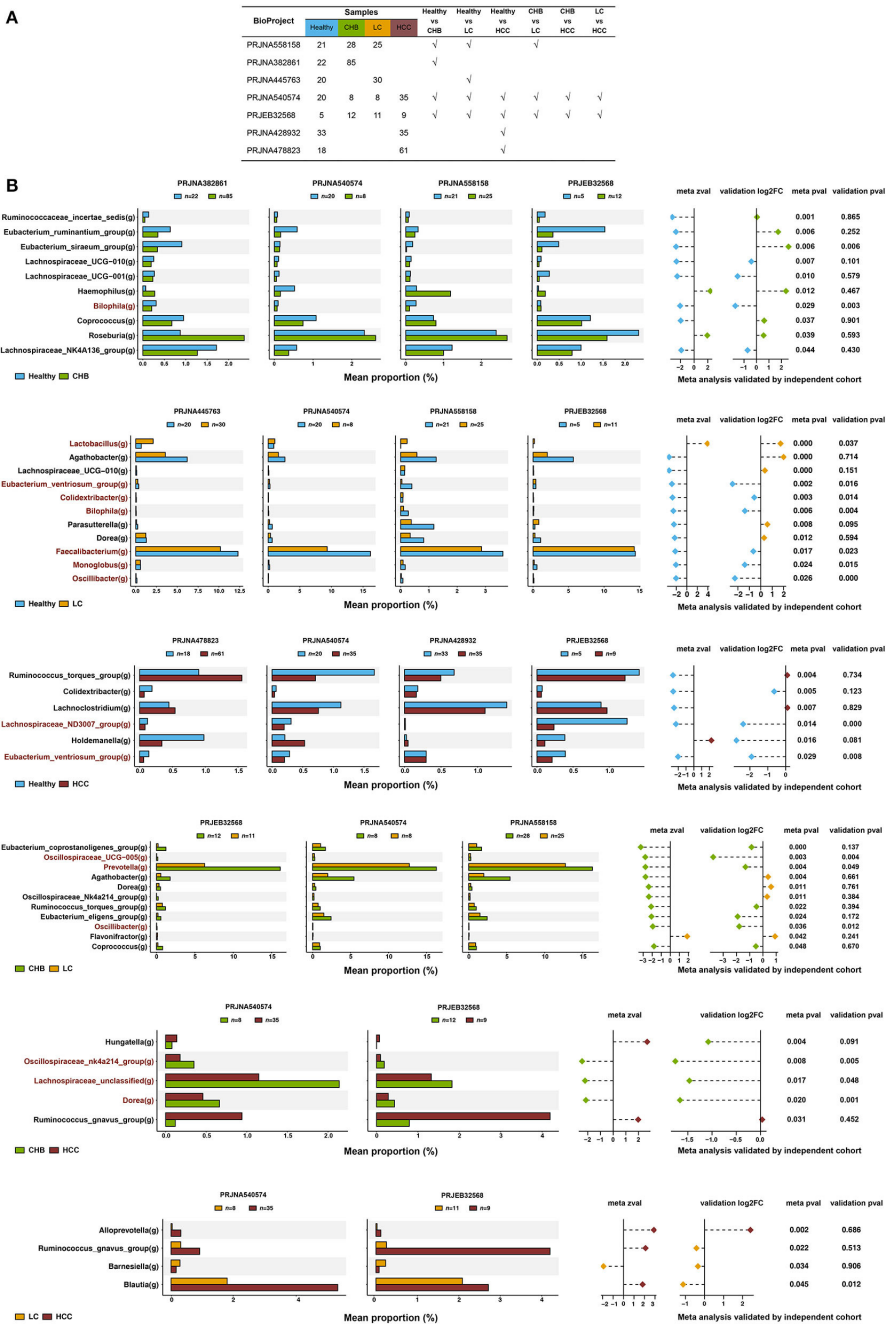
Signature microbiome taxa identified in the meta-analysis. **(A)** The public datasets included for each pair wise comparison. **(B)** Mean proportion of each genus between groups of interest in each individual dataset, and the summary statistics for the random effect meta-analysis for each pair wise comparison. The genus was highlighted by red if it was consistently and significantly altered in both public and validation datasets. Shown are the genera at the *p*-value threshold of 0.05.

Analysis of individual dataset revealed a significantly association of the microbiome beta diversity with disease subgroups for all datasets (Adonis *p* < 0.05), except for PRJNA428932 (Supplementary Figure S1). A total of 66 genera >0.01% in all seven public datasets was retained (Supplementary Table S3). For meta-analysis, different combinations of datasets were selected according to the 6 pair wise case-control comparisons (Figure 2A). For instance, for comparison between CHB and healthy controls, 4 studies (PRJNA558158, PRJNA382861, PRJNA540574, and PRJEB32568) involving these two groups were incorporated. The same strategy was applied to other case-control comparisons. The summary statistics of microbiome-disease association were synthesized between studies involving in the same comparison using random effect statistical meta-analysis.

A total of 35 genera were identified as differentially abundant in the pair wise comparisons between four disease stages (Figure 2B, *p* < 0.05). For example, 10 genera were identified as differentially abundant in the statistical meta-analysis between CHB and healthy controls. Among them, *Haemophilus* and *Roseburia* were increased in CHB; whereas the other 8 genera were decreased. About 11 genera were differentially abundant between LC and healthy controls, of which *Lactobacillus* was the only genus enriched in LC. Total six genera were differentially abundant between HCC and healthy controls, of which *Holdemanella* was the only genus increased in HCC. And 11 genera were differentially abundant between LC and CHB, of which only *Flavonifractor* was enriched in LC. Five genera were differentially abundant between HCC and CHB, of which *Hungatella* and *Ruminococcusgnavus_group* were enriched in HCC. Last, four genera were differentially abundant between HCC and LC, of which only *Barnesiella* was depleted in HCC.

**Figure 3:**
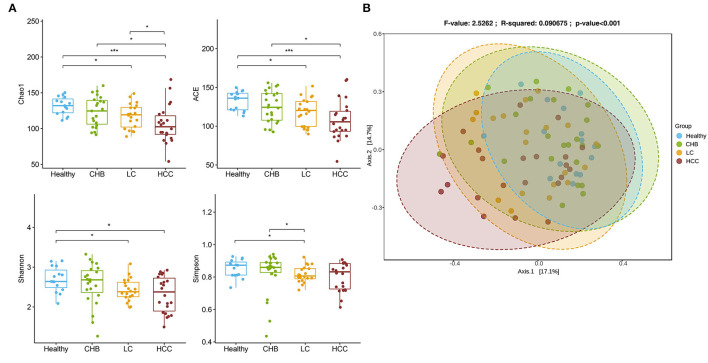
Overview of the gut microbiome in the validation cohort. **(A)** Alpha diversity for each disease status concluding Chao1, ACE, Shannon and Simpson. The differences were calculated by Wilcoxon test on genus level. *p < 0.05; ***p < 0.001. **(B)** Principal Coordinates Analysis (PCoA) of microbiome beta-diversity based on Bray-Curtis dissimilarity index.

To validate the above signatures, we collected fecal samples from 15 healthy controls, 23 CHB, 20 LC, and 22 HCC patients recruited at Panyu Central Hospital, Guangzhou, China, and analyzed their microbiomes. There was a continuous decreasing trend of alpha diversity in patients with enhanced disease stages (Figure 3A). Principal coordinate analysis based on Bray-Curtis dissimilarity showed that the microbiota was significantly associated with disease status (Adonis, *p* < 0.001), with HCC being most deviated from the other disease subgroups (Figure 3B).

The results of the meta-analysis were then compared to those of the validation cohort. For all 35 differential genera identified in the meta-analysis, 26 (74.3%) showed the same direction of alteration in the independent cohort, suggesting a reasonable level of consistency between the two datasets. Across them, 13 genera were significantly and consistently altered in the same direction in both public and validation datasets (*p* < 0.05, highlighted in red in Figure 2B). In comparison with healthy controls, *Bilophia* was significantly decreased in CHB; *Lactobacillus*was significantly increased in LC, whereas *Colidextribacter, Bilophila, Faecalibacterium, Monoglobus*, and *Oscillibacter* were significantly depleted; and *Lachnospiraceae_ND3007_group* and *Eubacterium_ventriosum_group* were significantly decreased in HCC. For comparison in between disease stages, *Oscillospiraceae_UCG*−*005, Prevotella*, and *Oscillibacter* were depleted in LC vs. CHB, and an unclassified *Lachnospiraceae* and *Dorea* were depleted in HCC vs. CHB (Figure 2B). No taxa were consistently depleted in HCC vs. LC at a *P*-value cutoff of 0.05. At a *P*-value cutoff of 0.1, *Ruminococcaceae_incertae_sedis, Clostridia_UCG-014, Oscillospiraceae_UCG-002* and *Coprococcus* were depleted in HCC vs. LC (Supplementary Figure S2).

**Figure 4:**
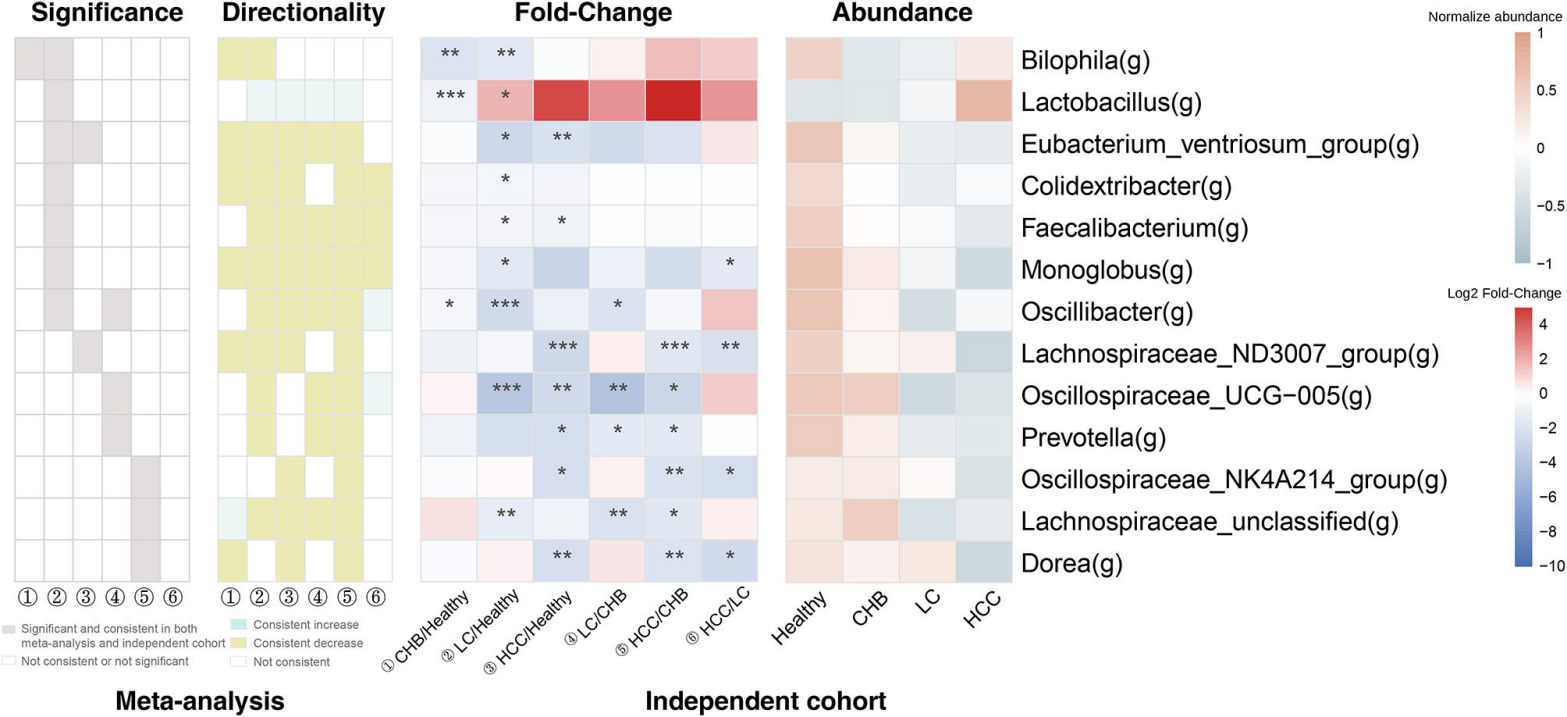
The consistency of the 13 signature genera between public and validation datasets. For each genus, the gray box indicates the pair wise comparison that the genus was both significantly and consistently altered in public and validation datasets. The green and yellow box indicates the pair wise comparisons that the genus was consistently increased or decreased in public and validation datasets (but not necessarily significant). The 2 heat maps indicate the fold-change across the pair wise comparisons and the average relative abundance across the four subgroups in the validation dataset. ① CHB vs. Healthy; ② LC vs. Healthy; ③HCC vs. Healthy; ④ LC vs CHB; ⑤ HCC vs. CHB; ⑥ HCC vs. LC. *p < 0.05; **p < 0.01; ***p < 0.001.

**Figure 5:**
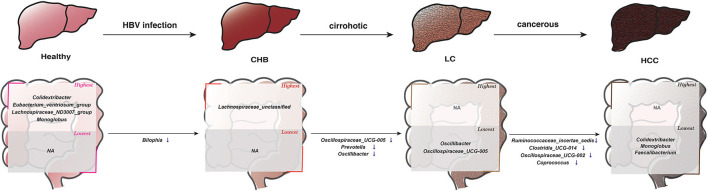
The consistent microbial signatures along the progression of the HBV-induced diseases based on results in this study. The genera in the “highest” box denote that their average relative abundances were most enriched in the corresponding subgroup compared to all other subgroups, whereas those in the “lowest” box represent that their abundance were most depleted in the corresponding subgroup. In the progression from 1 disease stage to another, ↑ denotes significantly increased abundance, ↓ denotes significantly decreased abundance.

It is worth noting that the majority of these 13 genera showed consistent direction of change between public and validation datasets across the six pair wise comparisons, indicating their robustness in association with the progression of HBV-induced liver disease (Figure 4; Supplementary Figure S3). Therefore, in addition to identifying microbial alteration in between disease stages, we were also able to derive signature taxa that were most enriched or depleted within each stage, based on the consistent signals. Specifically, *Colidextribacter, Eubacterium_ventriosum_group, Lachnospiraceae_ND3007_group* and *Monoglobus* were more elevated in controls; an unclassified *Lachnospiraceae* was most elevated in CHB, while no taxa were most depleted in these two groups. On the other hand, *Oscillibacter* and *Oscillospiraceae_UCG-005* were most depleted in LC, *Colidextribacter, Monoglobus*, and *Faecalibacterium* were most depleted in HCC, while no taxa were most enriched in these two groups (Figure 5).

**Figure 6:**
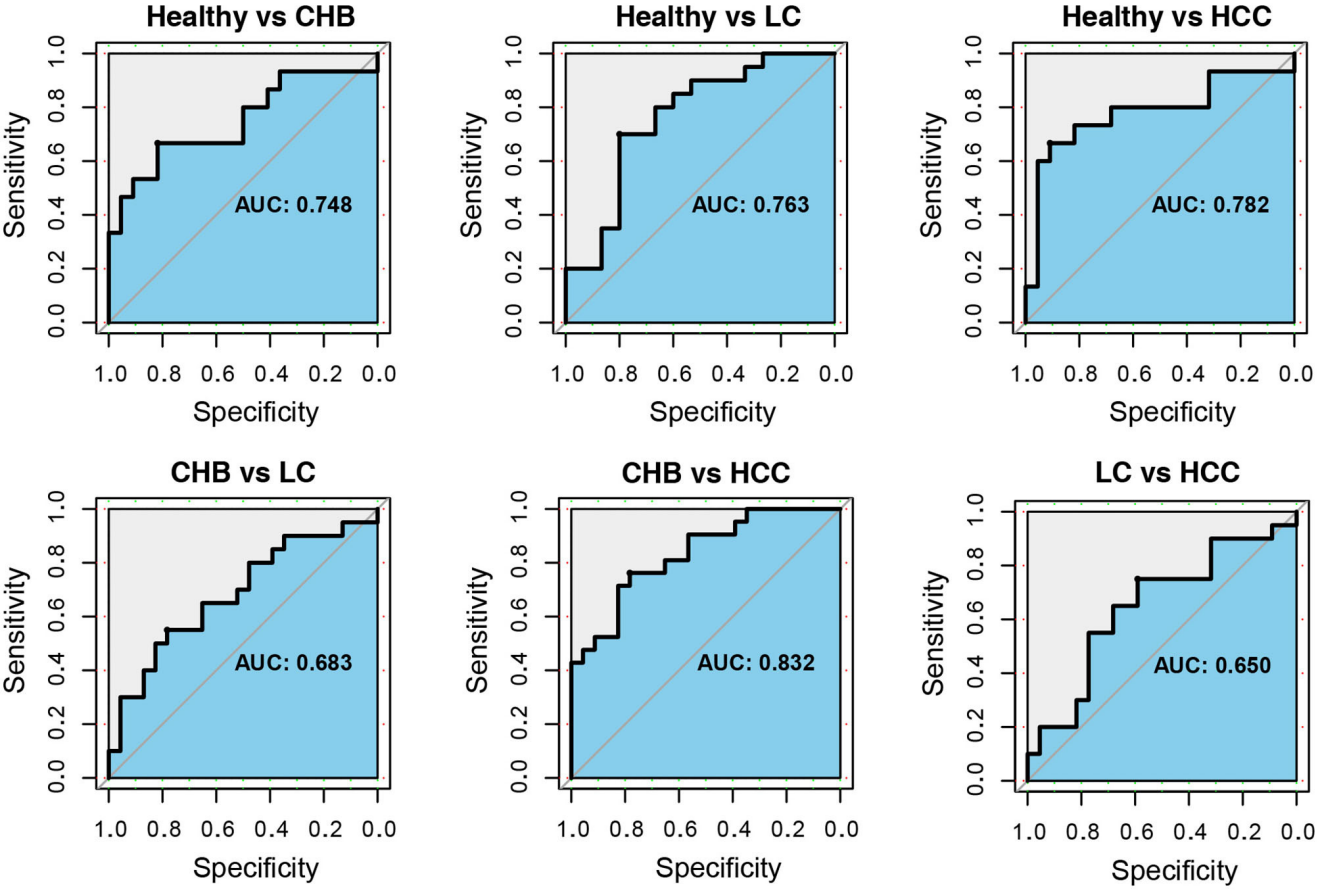
The receiver operating characteristic curves for classifiers based on the 13 signature genera. For each comparison between healthy and CHB, healthy and LC, healthy and HCC, CHB and LC, CHB and HCC, LC and HCC, the ROC curve and area under curve (AUC) were shown. The LASSO binary classifiers were trained by public dataset and validated in the independent cohort.

Finally, we tested the diagnostic power of these 13 genera. For each of the six pairwise comparisons among health and disease stages, a LASSO classifier was built using the 13 taxa. To test the generalizability, the classifier was trained using one public dataset and cross-validated in the validation cohort. In distinguishing CHB, LC and HCC from healthy controls, the areas under curve (AUCs) were 0.748, 0.763, and 0.782, respectively. In between disease stages, the AUCs were 0.683, 0.832, and 0.650 for CHB vs. LC, CHB vs. HCC and LC vs. HCC, respectively (Figure 6). The taxa with non-zero coefficients in each classifier were generally consistently identified as the signature taxa for each comparison (Supplementary Table S4; Figure 5).

## Discussion

Here, we present a comprehensive meta-analysis on public 16S rRNA datasets on HBV-induced liver diseases, which identified distinctive patterns of gut microbiome in association with the various disease stages. The meta-analysis enabled us to identify subtle microbial changes that were consistent across studies but may not reach significance within each individual study. The implementation of an independent validation cohort further increased the robustness of the identified taxa in association with disease progression.

HBV infection remains a prominent cause of hepatitis, liver cirrhosis, and HCC in the Asia-Pacific region, particularly in China (Sarin et al., 2020), as compared to the alcoholic or fatty liver disease driving liver cirrhosis and HCC in Western countries. Therefore, it is not surprising that the public datasets for HBV-induced liver diseases identified are all from China. We re-processed each dataset in a unified framework, particularly to account for the heterogeneity of the amplified hypervariable region across public datasets. As a unique strategy in our meta-analysis, different combinations of studies were employed for each pair wise comparison between groups, maximizing the sample sets that can be integrated for systematic meta-analysis.

We identified signature microbial taxa within each disease stage as well as taxa that were altered along the progression of different stages. Specifically, our data indicated a trend of depletion for key microbial taxa toward enhanced disease stages, suggesting continuous gut dysbiosis through disease progression. For instance, *Eubacterium_ventriosum_group, Monoglobus, Lachnospiraceae_ND3007_group*and *Colidextribacter* were most enriched in healthy individuals. *Eubacterium*and *Lachnospiraceae* are butyrate-producing bacteria and could have anti-inflammatory roles (Barcenilla et al., 2000). Specialized in pectin degradation, *Monoglobus* was shown to be negative associated with neutrophilic inflammation and severe liver injury (Kim et al., 2019; Chen et al., 2021; Zha et al., 2022). Dang et al. showed that decrease of *Eubacterium_ventriosum_group* and *Monoglobus* was associated with enhanced systemic inflammation (Dang et al., 2022). And *Colidextribacter* could modulate hepatic TLR4 and NF-κB signaling to reduce LPS-induced liver damage and inflammation (Mager et al., 2020; Guo et al., 2021). From healthy to CHB, *Bilophila* decreased significantly, whereas an unclassified *Lachnospiraceae* taxa was enriched. *Bilophila* was found as negatively associated with ALT and AST, suggesting its relevance to attenuated liver inflammation (Wang et al., 2021). From CHB to LC, *Oscillibacter, Oscillospiraceae_UCG-005*, and *Prevotella* were depleted. The decrease of gut *Oscillibacte*r and *Prevotella* was observed in severe alcoholic hepatitis and amyotrophic lateral sclerosis (Fang et al., 2016; Kim et al., 2021), while *Oscillospiraceae_UCG-005* was decrease inprimary sclerosing cholangitis (Liu et al., 2021). From LC to HCC, *Ruminococcaceae, Clostridia_UCG-014, Oscillospiraceae_UCG-002* and *Coprococcus* were decreased. And *Monoglobus* and *Colidextribacter, Faecalibacterium* were found to be most depleted in HCC. *Faecalibacterium* is commensal taxa whose decrease was found as a marker for early tumor establishment (Mangifesta et al., 2018). Likewise, *Coprococcus*was reported to decreased in patients with early-stage breast cancer or lung cancer (Liu et al., 2019; Bobin-Dubigeon et al., 2021). In addition, *Faecalibacterium* and *Coprococcus* are both capable of producing butyrate, whose depletion mayaffect gut permeability, increasing bacterial translocation in favor of cancer. Furthermore, classifiers built using these taxa showed diagnostic power in between health and all disease stages, implicating the potential of the gut microbial taxa as non-invasive biomarkers for diagnosis of pan-HBV-induced liver disease.

Our study has limitations. First, although we took efforts to address heterogeneity in 16S rRNA hypervariable region, other confounding effects from experimental procedures, sequencing platform scan not be unambiguously addressed. There is no optimal solution to fully account for the inter-study batch effects for microbiome data (Gibbons et al., 2018; Wang and LeCao, 2020). Nevertheless, it should be noted that, rather than directly concatenating the raw datasets, we chose to analyze each study separately and pool summary statistics from each dataset using a random effect model, which is a more conservative approach but effective in reducing data heterogeneity (Ramasamy et al., 2008). Secondly, all public and validation datasets are cross-sectional. Although somewhat challenging, longitudinal cohorts that monitor the disease trajectories from CHB, LC to HCC would be valuable to validate our findings and help interrogate potential causality between microbiome and liver diseases. Third, additional clinical parameters such as HBV carrier stage and HBV load are required to refine our observations, by identifying taxa associated with disease severity. Fourth, although the diagnostic power of classifiers built using the identified taxa was decent when comparing healthy status and diseases, the AUCs were relatively modest in between different disease stages, a result possibly due to limited sample size. The results therefore need to be validated in further larger cohorts preferably with different demographic backgrounds.

In summary, we identified microbial signatures along the progression of HBV-induced liver disease. These results may help identify candidate, non-invasive microbiome biomarkers that indicate disease progression and inform future mechanistic studies on the role of gut microbiome in HBV-induced liver diseases.

## Data Availability Statement

The raw 16S rRNA gene sequencing reads presented in this study are deposited in National Center for Biotechnology Information (NCBI), under accession number PRJNA838083.

## Ethics Statement

The studies involving human participants were reviewed and approved by Ethics Review Committee of the Guangzhou Panyu Central Hospital (Permit Number ID: PYRC-2021-115). The patients/participants provided their written informed consent to participate in this study. Written informed consent was obtained from the individual(s) for the publication of any potentially identifiable images or data included in this article.

## Author Contributions

RL and XY analyzed and interpreted the data and wrote the manuscript. JY, ZZ, and YiW collected the public data. XL and XH processed the fecal samples. YuW and XF collected the fecal samples. WS, WZ, and ZW participated on study design and revised the manuscript. All authors read and approved the final version of the manuscript.

## Funding

This work was supported by the National Natural Science Foundation of China (31970112, 32170109, and 41907211), and the Science and Technology Foundation of Guangdong Province (2019A1515011395).

## Conflict of Interest

The authors declare that the research was conducted in the absence of any commercial or financial relationships that could be construed as a potential conflictof interest.

## Publisher's Note

All claims expressed in this article are solely those of the authors and do not necessarily represent those of their affiliated organizations, or those of the publisher, the editors and the reviewers. Any product that may be evaluated in this article, or claim that may be made by its manufacturer, is not guaranteed or endorsed by the publisher.
