# Supplementary figures and images for: Gut Microbiome Signatures in the Progression of Hepatitis B Virus-Induced Liver Disease
Source: Front Microbiol. 2022 Jun 6;13:916061. doi: 10.3389/fmicb.2022.916061 (PMC9208012; doi:10.3389/fmicb.2022.916061)

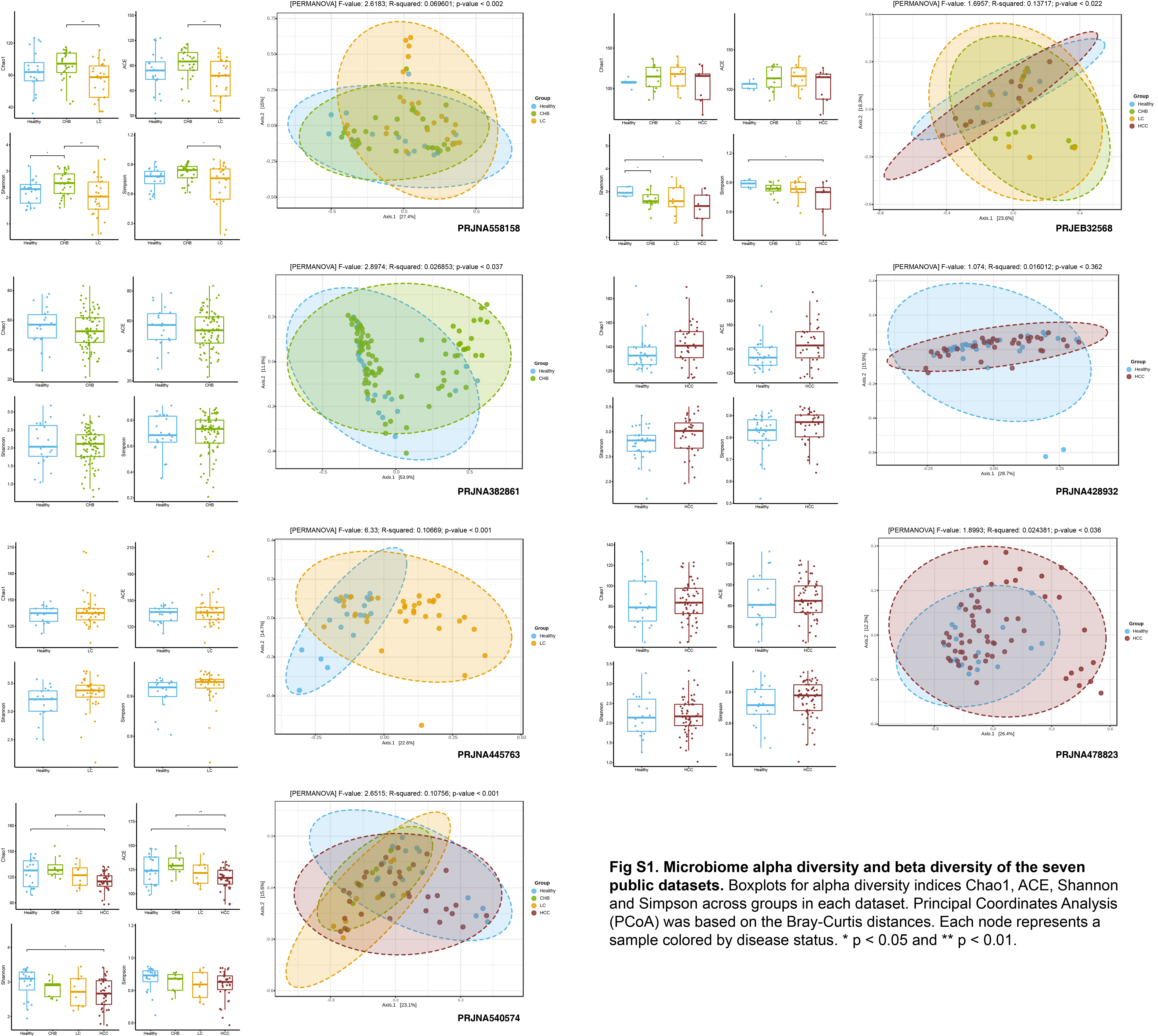

Supplement: Supplementary file 5 [file Image_1.jpeg]

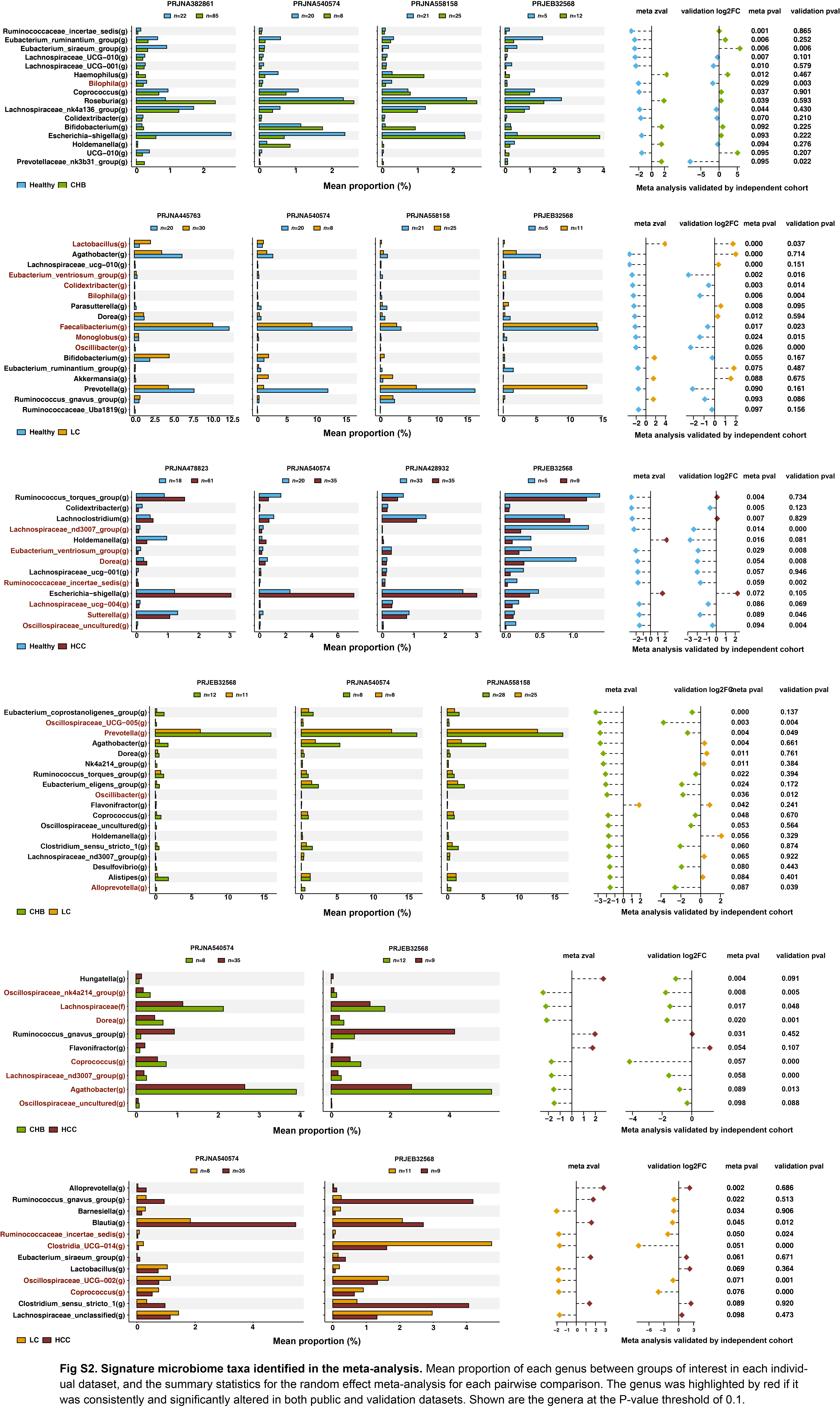

Supplement: Supplementary file 6 [file Image_2.jpeg]

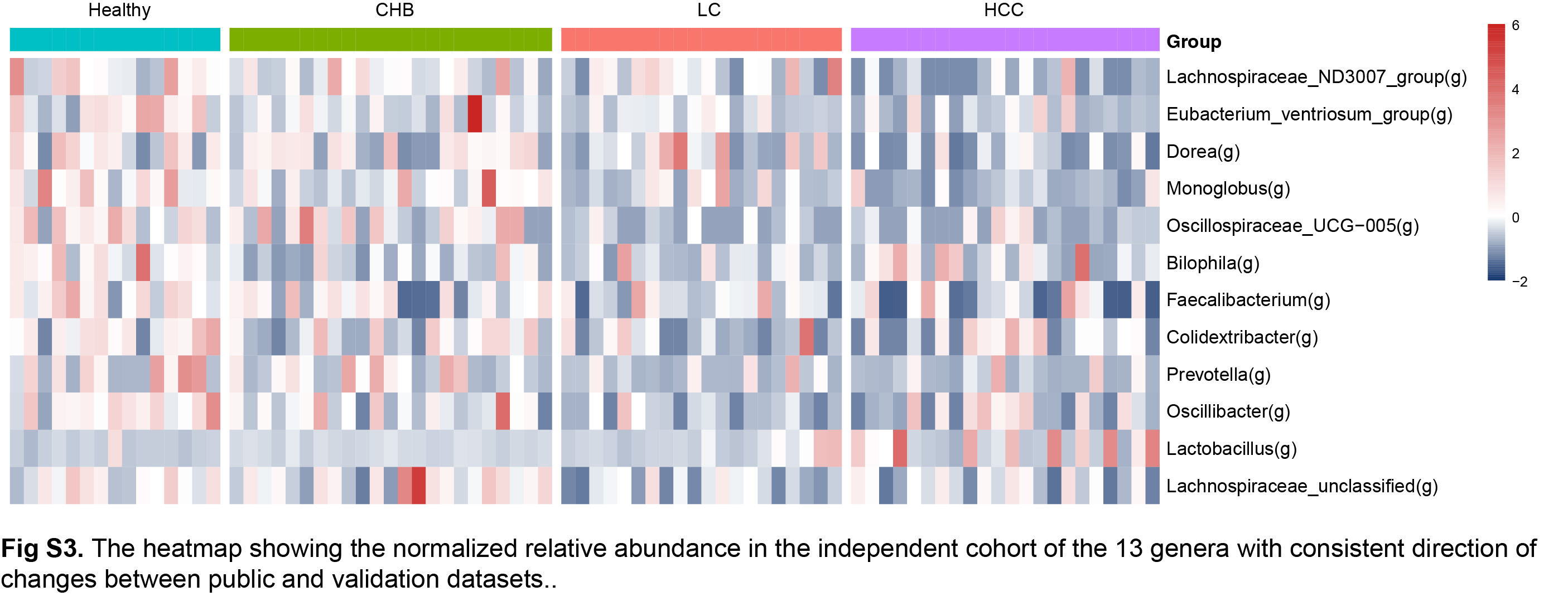

Supplement: Supplementary file 7 [file Image_3.jpeg]
